# To Make Joints Tight with Amalgam

**Published:** 1893-08

**Authors:** W. S. Simonton

**Affiliations:** Cameron, W. VA.


### TO MAKE JOINTS TIGHT WITH AMALGAM.

BY DR. W. S. SIMONTON, CAMERON, W. VA.

In amalgam fillings the union of the amalgam and cavity walls must be close and tight to make a reliable filling. The line of contact must be such that the secretions of the mouth, or any substance whatever, cannot pass between the walls of the cavity and the body of the filling. In fact, this result must be obtained with whatever material may be used for the filling. Generally amalgam does not form so good a joint, if this term may be used, nor maintain it so well, as some other materials; and here, I think, is one of the causes for the many failures of amalgam fillings. No standard amalgam, properly manipulated, is porous, and if any substance enters a cavity filled with it and causes decay, it must do so through the joint or line of contact of the material and tooth substance. Hence this perfect union must be obtained and preserved if good results are to be secured with amalgam fillings. In teeth of good structure, and a crown cavity not too large, these results are secured by filling in the ordinary way. In teeth soft and friable, and in approximal cavities, it is especially hard to maintain, and the tooth will soon be found decaying along the line of contact of metal with cavity walls. Fillings of this kind are always, or almost always, failures; hence the importance of obtaining and maintaining this perfect junction between the two substances. There is an old mechanical resort of introducing a third and different body between two other bodies to assist in joining them, as gum packing in steam- or water-joints or glue between two pieces of wood.

In amalgam fillings, having prepared the cavity, wash it with alcohol and disinfect, and, having prepared the amalgam by triturating with alcohol in a small mortar, prepare some thin cement,—just how thin I know of no way of stating, but will refer again to this. Having the cement prepared, with the rubber dam on, line the edges of the cavity with the thin cement by taking a small quantity on a thin, narrow instrument, and scraping it off on the edges of the cavity entirely around the outer edge, so that the cement will hang over in the cavity. Then introduce the amalgam as nearly as possible into the centre and work from this point, and press it out against the walls of the cavity. Press it with force sufficient to carry it to the walls. This will leave an open space in the centre of the incomplete filling, into which insert more amalgam, pressing it in with considerable power, so that it will back up and join with the amalgam already pressed out against the walls. It will readily be seen that the cement must be so thin that it can be forced away, allowing the amalgam to approach the walls. After filling the cavity and pressing and condensing thoroughly, remove the surplus, and the filling will generally be found hard enough to take a good finish. The work of filling and pressing to the walls must be done quickly, as the cement will very soon commence to harden and not allow the amalgam to join the walls, leaving small portions that will soon wear away and produce a leak or expose a small space to decay. This is not mixing cement and amalgam to form a filling-material, but is using the cement to secure a close or impervious union or joining of the two different substances. The cement is, of course, not entirely pressed out; a small portion becomes mixed with the amalgam at the border and glues it to the walls of the cavity, so as to cause it to adhere very closely; yet the portion of cement remaining, if the operation be properly performed, is so divided up by the amalgam and protected by it that it cannot wear or wash away, but still performs its part by maintaining the integrity of the line of contact.

This is the best method of making amalgam fillings I have ever tested, especially, as I stated previously, if the filling be large or approximal. It is particularly serviceable in the teeth of the young,—much more so if it is desirable to make a filling that will wear longer than cement. I began this method five years ago, and filled the teeth of young persons where I would otherwise have used cement, and find the filling good at the present time. I was performing some work for a young woman for whom, when a child, I had filled a first lower molar by this method. She had called to have the tooth extracted, as it was badly decayed and broken down, crown almost gone, there being far more of the filling left than the tooth crown. When I examined it, the filling was in good order, no decay about the edges, they being well joined up and preserving the tooth, or what there was of it, two-thirds of the crown being built up of amalgam.

Amalgam is generally decried as a filling-material, yet it is one of the sheet-anchors in preserving teeth, and does this better than gold or any other filling-material, perhaps more than all others together. Yet there are occasions when it fails from want of care on the part of the operator. Amalgam fillings should be as well and as carefully made as when other material is used. This method will often help out of a difficult place, and will under nearly all circumstances give better results. To the dentist who will not stoop to amalgam, and uses nothing but gold, it will be of no value; but to others, who cannot fill every cavity that is presented with gold, or who are willing to save a tooth even with amalgam, it may help.

